# Strong quantum scarring by local impurities

**DOI:** 10.1038/srep37656

**Published:** 2016-11-28

**Authors:** Perttu J. J. Luukko, Byron Drury, Anna Klales, Lev Kaplan, Eric J. Heller, Esa Räsänen

**Affiliations:** 1 Nanoscience Center, Department of Physics, University of Jyväskylä, Jyväskylä FI-40014, Finland; 2 Department of Physics, Tampere University of Technology, Tampere FI-33101, Finland; 3 Department of Physics, Massachusetts Institute of Technology, Cambridge, Massachusetts 02139, USA; 4 Department of Physics, Harvard University, Cambridge, Massachusetts 02138, USA; 5 Department of Physics and Engineering Physics, Tulane University, New Orleans, Louisiana 70118, USA

## Abstract

We discover and characterise strong *quantum scars*, or quantum eigenstates resembling classical periodic orbits, in two-dimensional quantum wells perturbed by local impurities. These scars are not explained by ordinary scar theory, which would require the existence of short, moderately unstable periodic orbits in the perturbed system. Instead, they are supported by classical resonances in the unperturbed system and the resulting quantum near-degeneracy. Even in the case of a large number of randomly scattered impurities, the scars prefer distinct orientations that extremise the overlap with the impurities. We demonstrate that these preferred orientations can be used for highly efficient transport of quantum wave packets across the perturbed potential landscape. Assisted by the scars, wave-packet recurrences are significantly stronger than in the *unperturbed* system. Together with the controllability of the preferred orientations, this property may be very useful for quantum transport applications.

Quantum scars^1^ are enhancements of probability density in the eigenstates of a quantum chaotic system that occur around short unstable periodic orbits (POs) of the corresponding classical system. Scars have been observed experimentally in, e.g., microwave cavities^2^,^3^, optical cavities^4^,^5^, and quantum wells^6^,^7^, and computationally in, e.g., simulations of graphene flakes^8^ and ultracold atomic gases^9^.

Before the existence of scars was reported by Heller^10^, eigenstates of a classically chaotic system were conjectured to fill the available phase space evenly, up to random fluctuations and energy conservation. If high-energy eigenstates of non-regular (i.e., generic) systems were indeed featureless and random, controlled applications in that regime would be difficult. Scars are therefore both a striking visual example of classical-quantum correspondence away from the usual classical limit, and a useful example of a quantum suppression of chaos.

In this work we describe quantum scars present in otherwise separable systems disturbed by local perturbations such as impurity atoms. In this case, scars are formed around POs of the corresponding *unperturbed* system. The scars are strikingly strong and common, and they have properties that cannot be explained by ordinary scar theory. Instead, we show that the symmetry of the unperturbed system and the local nature of the perturbations give rise to a new scarring mechanism.

In the following, values and equations are given in natural units where the quantum Hamiltonian is simply 
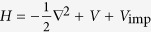
, where *V* is the unperturbed potential and *V*_imp_ represents the perturbation. The eigenstates of *H* are solved with imaginary time propagation in real space^11^.

## Model system

The scarring mechanism, explained later in this article, is very general; it requires only that the unperturbed system is separable, and that the perturbing impurities are sufficiently local. In the following we focus, for simplicity, on a few prototypical examples of a circularly symmetric, two-dimensional potential well *V*(*r*) perturbed by randomly scattered Gaussian bumps.

The classical POs of any circularly symmetric *V*(*r*) can be enumerated directly (see Supplementary Section S1 and ref. 12). Each PO is associated with a resonance, where the oscillation frequencies of the radial and angular motion are commensurable. The PO structure is especially simple if *V*(*r*) is a homogeneous function (i.e., *V*(*r*) ∝ *r*^*a*^), since then POs with different total energies differ only by a scaling, i.e., the shape of POs does not depend on the energy.

In the following we use *a* = 5. Its shortest non-trivial PO, i.e., besides the circular orbit and the case with zero angular momentum, is a five-pointed star, which is both easily distinguishable and short. This PO corresponds to a 2 : 5 classical resonance, where the orbit circles the origin twice in the time of five radial oscillations. The case of *a* = 2, the harmonic oscillator, is special and ill-suited for drawing general conclusions, while for *a* = 1, 3, 4 the shortest non-trivial POs are longer.

**Figure 1:**
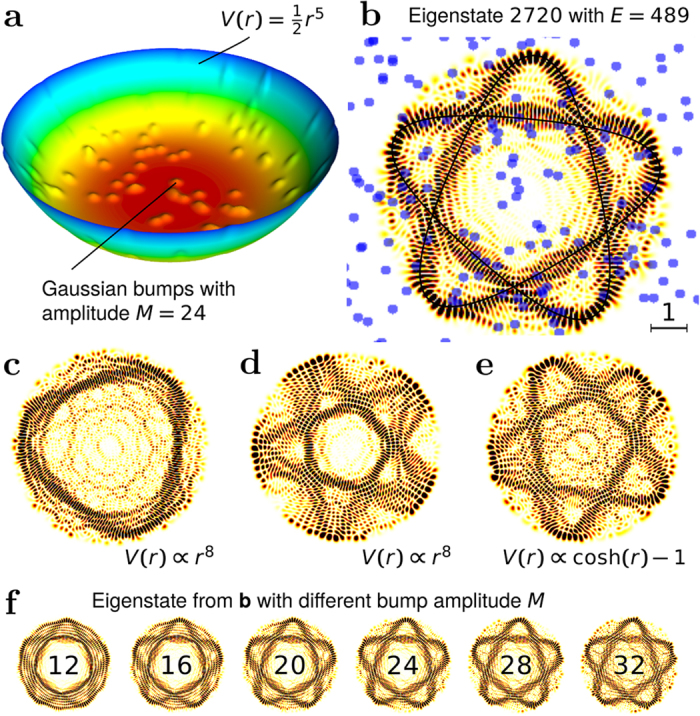
Examples of scarred eigenstates in perturbed potential wells. An example potential well 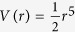 perturbed by Gaussian bumps is shown in (**a**) and one of its strongly scarred eigenstates is shown in (**b**). Blue markers denote the locations and full widths at half maximum of the bumps, and the corresponding PO of the unperturbed potential is drawn as a solid line. Note that several bumps are located on the scar path. Examples of scars in other potentials are shown in (**c–e**). The eigenstate in (**b**) at different bump amplitudes *M* is shown in (**f**). The scar shape and orientation remain unchanged as *M* increases.

For easier comparison between the results, we focus in the following on a single example shown in Fig. 1(a), where Gaussian bumps with amplitude *M* = 24 are distributed in an unperturbed potential 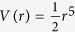. The average density of the bumps is two per unit square, and thus for a typical energy of 500, approximately a hundred bumps exist in the classically allowed region. The full width at half maximum (FWHM) of the Gaussian bumps is 0.235, which is similar to the typical local wavelength of the eigenstates we consider.

The amplitude of the bumps is small compared to the total energy, making each individual bump a small perturbation. Nevertheless, together the impurities are sufficient to destroy classical long-time stability; any stable structures present in the otherwise chaotic Poincaré surface of section are tiny compared to *ħ* = 1.

## Scar observations

Figure 1(b) shows an example of a scar found in the eigenstates of the example potential described previously. In this case approximately 80% of the probability density resides on the star path. Figure 1(c–e) show examples of scars in a homogeneous potential with exponent *a* = 8, and in a non-homogeneous potential *V*(*r*) ∝ cosh(*r*) − 1. For more discussion about scars in other potentials please see Supplementary Sections S3 and S4. In all cases the scars follow POs in the corresponding unperturbed system. We emphasise that the strong scars are not a rare occurrence; for the potential in Fig. 1(a) over 10% of all eigenstates are clearly scarred by the five-pointed star orbit.

In ordinary scar theory^10^, each scar corresponds to a moderately unstable PO in the classical system. In this case such orbits do not exist. For example, the shortest and least unstable PO near the scar shown in Fig. 1(f) for *M* = 16 closes on itself after two rounds around the scar, and has a one-period stability exponent^13^ *χ* ≈ 5. In linear scar theory^1^,^14^ this is too unstable to cause observable scarring, since scarring strength falls of exponentially with *χ*, although nonlinear contributions from homoclinic orbits (HOs)^15^,^16^,^17^ could conceivably increase the scarring strength in some cases assuming lucky constructive interference between many HOs.

Ordinary scar theory is more firmly excluded by the behaviour of the scars as a function of the bump amplitude *M*. If *M* is increased while keeping *V*_imp_ otherwise unchanged, the scars grow stronger and then fade away without changing their orientation, as shown in Fig. 1(f). With or without nonlinear contributions from HOs, a scar caused by ordinary scar theory would become rapidly weaker, since the stability exponent of a PO increases with *M*.

Comparing scars at different energies *E* also reveals that they occur in only a few distinct orientations, and these orientations change quite slowly with *E*. Later in this manuscript this stability is quantified by wave-packet methods. For the example *V*_imp_ used here there are three preferred orientations. For other impurity realisations the number and location of the preferred orientations vary, but the existence of preferred scar orientations is a generic feature (see Supplementary Section S5). This feature is also not explained by ordinary scar theory, as the existence and stability of POs should be very sensitive to changes in *E*.

We also note that the scars are not explained by dynamical localization^18^,^19^. Although present in systems discussed here, dynamical localization corresponds to localization in angular momentum space, whereas the scars are localized in position space. Dynamical localization also does not explain the preferred orientations.

## Wave-packet analysis

Gaussian wave packets are a standard tool for studying eigenstate scarring^10^. If a Gaussian wave packet |*ϕ*〉 is centred on a scarring PO, its autocorrelation function *A*(*t*) = 〈*ϕ*(0)|*ϕ*(*t*)〉 shows clear short-time recurrences with a period *T* matching the period of the PO. In the linear theory of ordinary scarring the strength of these recurrences |*A*(*nT*)|^2^ dies out as 1/cosh(*χn*), where *χ* is the stability exponent of the PO^14^. In addition, |〈*ϕ*(0)|*ψ*〉| is large for an eigenstate |*ψ*〉 scarred by the particular PO, making wave packets useful for picking out eigenstates scarred by a particular PO (see Supplementary Section S2).

**Figure 2:**
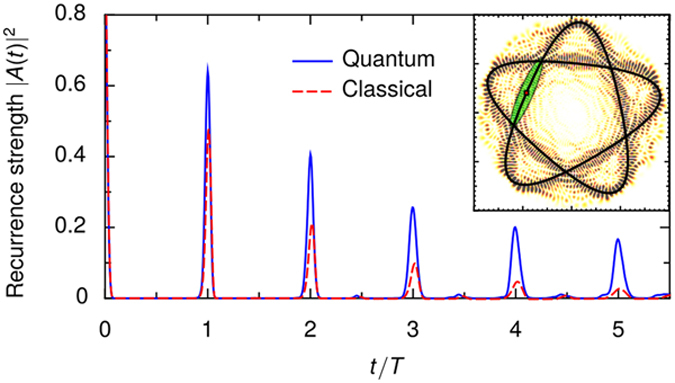
Recurrence strength of a Gaussian wave packet propagating along the scar in Fig. 1(b). The quantum recurrence strength (solid blue line) is compared with the corresponding classical recurrence strength (dashed red line). Time is shown in units of the period *T* of the unperturbed PO. The scar, the corresponding PO (solid black line) and the location and full width at half maximum of the initial Gaussian (green area) are shown in the inset.

In our case we use the PO of the unperturbed system to initialise the wave packet. The energy and the orientation of the PO are matched to the scarred eigenstate. The width parameters of the Gaussian are matched approximately to the geometry of the scar (see the inset in Fig. 2).

The wave packets selected in this way have a considerable energy uncertainty, so that many scars, sharing the same approximate orientation, contribute to the recurrences. A typical FWHM of the wave packet is approximately 50 energy units, or 400 eigenstates.

Figure 2 shows the recurrence strength |*A*(*t*)|^2^ for a wave packet travelling on the scar shown in Fig. 1(b). Clear periodic recurrences are visible, with a period that matches the period of the unperturbed PO.

To account for purely classical effects, we compare the results with the recurrence of the corresponding classical density. This is given by the Wigner transform *G* of the wave packet. Its recurrence strength was calculated by sampling 80 000 classical initial states {(**r**_*i*_, **p**_*i*_)}_*i*_ from the distribution *G*, propagating each in time, and computing 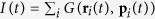. Classical time integration was performed with the sixth-order symplectic integrator of Blanes & Moan^20^. Once normalised so that *I*(0) = 1, *I*(*t*) corresponds to the quantum recurrence strength. The classical recurrences are significantly weaker than the quantum recurrences even at *t* = *T*, and this difference grows rapidly at later times, illustrating the quantum nature of the phenomenon.

**Figure 3:**
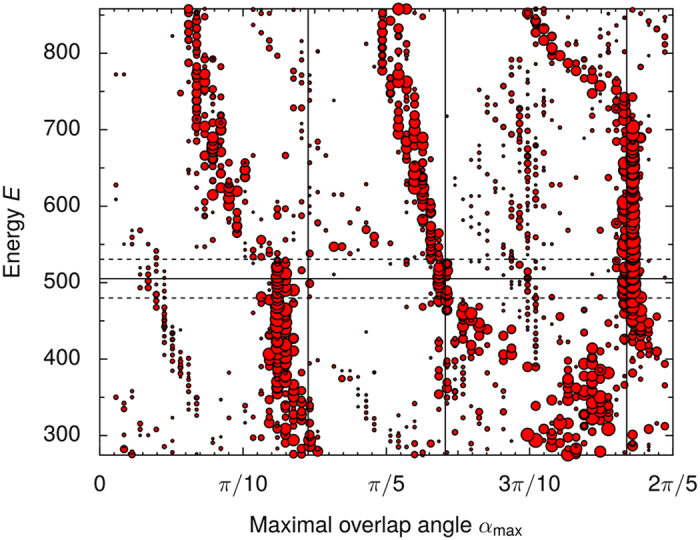
Scatter plot demonstrating the existence and stability of the preferred scar orientations. For each eigenstate, a circle is marked on the orientation angle *α* showing the highest overlap between the eigenstate and an initial Gaussian wave packet with the given orientation. The radius of the circle gives its squared magnitude. Squared overlaps of less than 3 ⋅ 10^−3^ are excluded. The vertical coordinate is the common energy of the eigenstate and the PO. The solid and dashed horizontal lines show, respectively, the mean energy and energy FWHM of the wave packet used in Fig. 2. For easier comparison, orientation angles with peaks at *t* = 4*T* in Fig. 4 are marked with solid vertical lines.

The existence of stable preferred scar orientations can be demonstrated by systematically detecting scarred states with wave packets. Figure 3 shows how the overlap of the initial wave packet with the target eigenstate depends on the energy of the eigenstate and the orientation of the PO used to initialise the wave packet. The orientation coordinate *α* is such that at *α* = 0 the wave packet starts on the positive *y*-axis and heads to the right.

In an angular window of 2*π*/5 (after which the PO is the same), three branches of high overlaps are visible, corresponding to the preferred orientations. Note that the rightmost branch is roughly vertical for *E* = 400 …700, corresponding to an increase of the average radius of the PO by roughly 0.3 units, which is the length scale of the individual bumps.

**Figure 4:**
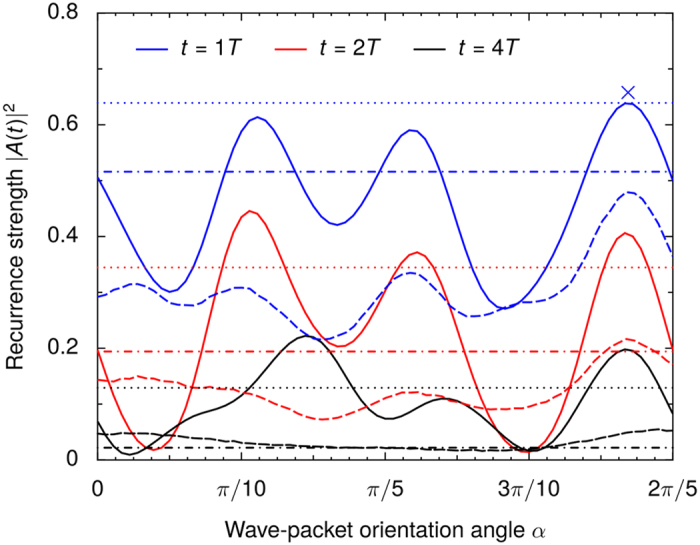
Amplitude of recurrence peaks shown in Fig. 2 as a function of the initial wave-packet orientation *α*. Solid lines and dashed lines show the quantum and classical results, respectively. Dash-dotted and dotted lines show the quantum and classical results in the unperturbed system, respectively. Blue, red, and black lines correspond to snapshots after one, two, and four periods, respectively. The orientation angle used in Fig. 2 is marked with a blue cross. This is also the orientation that matches the scar shown in Fig. 1(b).

Figure 4 shows how the amplitude of the recurrence peaks in Fig. 2 depend of the orientation angle *α*. Both quantum and classical wave-packets show stronger short-time recurrences at the preferred orientations, indicating that the preferred orientations can also be explained classically. However, especially at the preferred orientations, quantum late-time recurrences are much stronger than in the classical case.

Note that the quantum recurrences are stronger than the classical ones even on average, suggesting that there is also an effect that strengthens quantum recurrences at all orientations.

To highlight how strong the quantum recurrences are, a comparison to recurrences in the unperturbed system is also shown in Fig. 4. For the preferred orientations, the quantum late-time recurrences greatly exceed the strength of both the quantum and the classical recurrences in the *unperturbed* system. Via the creation of strong scars with stable preferred orientations, the randomly scattered impurities *enhance* the coherent propagation of quantum wave packets in the potential well!

## Perturbation Theory

Both the existence of scars and the preferred orientations can be explained by perturbation theory. This explanation is based on two ingredients. Firstly, special nearly-degenerate subspaces exist in the basis of unperturbed eigenstates. Secondly, the local perturbations select scarred eigenstates from these subspaces.

The eigenstates of the unperturbed, circularly symmetric system are labelled by two quantum numbers (*r*, *m*), corresponding to radial and angular motion, respectively. States (*r*, ±*m*) are exactly degenerate, but in addition there are near-degeneracies that correspond to classical POs.

By the Bohr–Sommerfeld quantisation condition (a good approximation at high quantum numbers) the energy difference from increasing *r* or *m* by one is proportional to the classical oscillation frequency of the corresponding action. Thus, if a state (*r*, *m*) is nearby in action to a classical PO with a ratio *a* : *b* between the oscillation frequencies, the state (*r* + *a*, *m* − *b*) will be nearby in energy. The smaller *a* and *b* are the closer the near-degeneracy is. This creates “resonant sets” of unperturbed basis states, and a part of a resonant set can be almost degenerate. Expanding in the unperturbed basis reveals that the scarred eigenstates are localised to such near-degenerate subspaces.

A superposition of two resonant states will exhibit beating in both the radial and angular directions. Because the ratio of the beat frequencies is also *a*:*b*, the interference pattern will trace out the shape of the classical PO. Adding more resonant states with appropriate phases will narrow the region of constructive interference and sharpen the scar, and even a few basis states can create a distinctly classical-looking linear combination.

Similar reconstruction of classical-like states from (nearly) degenerate basis states has also been studied previously^21^,^22^,^23^,^24^. However, to create a lot of strongly scarred eigenstates with preferred orientations a mechanism that favours these scarred linear combinations is required.

Within the impurity strength regime that results in the strongest scarring, the perturbation *V*_imp_ couples predominantly only states that are nearly degenerate. One can therefore approximate the perturbed eigenstates by degenerate perturbation theory (DPT), i.e., by diagonalising *V*_imp_ within the near-degenerate subspace of resonant states.

The effect of non-resonant states that are nearly degenerate by chance is mitigated by the weak coupling of states with very different quantum numbers. The product of the wave functions of two such states oscillates rapidly even on the length scale of a single perturbation bump. As a result, the coupling term 〈*ψ*_1_|*V*_imp_|*ψ*_2_〉 decreases rapidly as a function of the difference in quantum numbers between the states |*ψ*_1_〉 and |*ψ*_2_〉.

The DPT-produced eigenstates corresponding to the extremal eigenvalues of *V*_imp_ are, by the variational principle, the states |*ψ*〉 that extremise the expectation value 〈*V*_imp_〉: = 〈*ψ*|*V*_imp_|*ψ*〉. This quantity for a non-scarred, spatially delocalised state is essentially an average value of *V*_imp_ over the entire accessible region, and never an extremum. Compared to the non-scarred states, the scarred states in a near-degenerate subspace have their probability amplitude concentrated in a much smaller region of space. For a *V*_imp_ consisting of local impurities the state that maximises (minimises) 〈*V*_imp_〉 will therefore generally be a scarred state oriented so as to coincide with an anomalously many (few) impurities.

By the previous argument the scar orientations are mostly selected by the positions of the impurities. Since the inner and outer radii of the POs change slowly with energy, the orientations that extremise 〈*V*_imp_〉 will be determined largely by the same impurities for many different resonant sets. This is the origin of the stability of the preferred orientations seen in Fig. 3.

**Figure 5:**
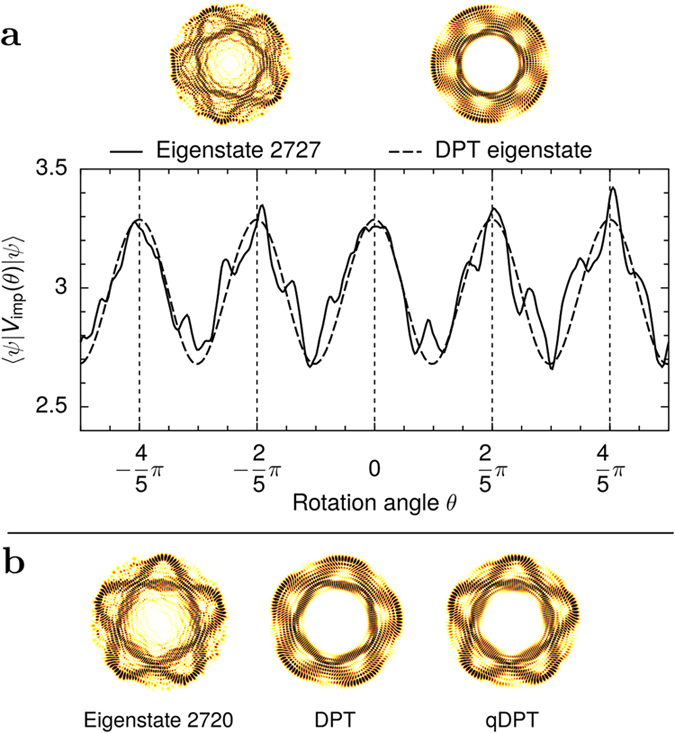
Reconstructing scarred eigenstates with degenerate perturbation theory (DPT). DPT reconstruction of a scarred eigenstate 2727 is shown in (**a**). The plot depicts the expectation value 〈*V*_imp_(*θ*)〉, rotated by angle *θ* from the original *V*_imp_, for both the actual eigenstate (solid line) and the eigenstate as predicted by DPT (dashed line). The plot shows that the orientation of the scar is such that 〈*V*_imp_〉 is maximised. The overlap between the true eigenstate and the DPT reconstruction is 56%. The DPT reconstruction is calculated from a resonant set of only three nearly degenerate basis state doublets. Similar reconstruction of the eigenstate 2720, shown also in Fig. 1(b), is shown in (**b**). The DPT reconstruction produces a scar, but not at exactly the correct orientation. An improved quasi-DPT approximation, which takes into account the imperfect degeneracy, corrects it. The overlap between the eigenstate and the reconstruction is 60% for DPT and 68% for qDPT. The DPT reconstruction uses three nearly degenerate doublets, while the qDPT reconstruction uses five.

Though a simple DPT approximation does not reproduce the true eigenstates exactly, it both explains the scarring and predicts the orientations of the observed scars well, as illustrated in Fig. 5. Taking into account the imperfect degeneracy amounts to diagonalising the full Hamiltonian instead of only the perturbation^25^. This means that in the matrix that is diagonalised the diagonal elements are shifted by the (small) spacings of the near-degenerate basis states. This “quasi-DPT” (qDPT) approximation improves the agreement between the DPT approximation and the exact eigenstates, as shown in Fig. 5(b).

## Summary and Outlook

To summarise, we have shown that a new type of quantum scarring is found in separable systems perturbed by local impurities. The scars are very common, and they tend to occur in discrete preferred orientations. This allows wave packets to propagate through the perturbed system with higher fidelity than in the unperturbed system.

The theoretical basis of the scarring is very general, requiring only classical resonances and local perturbations. Therefore the scarring should have consequences well beyond the simple models discussed here.

The implications of the enhanced wave packet recurrences for quantum transport will be an important area for future work. In an experiment, local perturbations similar to the ones used here could be generated by a conducting nanotip^26^,^27^,^28^, selecting particular scar orientations and enhancing the local conductance in a controlled way.

## Additional Information

**How to cite this article**: Luukko, P. J. J. *et al*. Strong quantum scarring by local impurities. *Sci. Rep*. **6**, 37656; doi: 10.1038/srep37656 (2016).

**Publisher's note:** Springer Nature remains neutral with regard to jurisdictional claims in published maps and institutional affiliations.

## Supplementary Material

Supplementary Information
