# Supplementary material for: Strong quantum scarring by local impurities
Source: Sci Rep. 2016 Nov 28;6:37656. doi: 10.1038/srep37656 (PMC5124902; doi:10.1038/srep37656)
Supplement: Supplementary Information [file srep37656-s1.pdf]

Strong quantum scarring by local impurities

Supplementary Information

Perttu J. J. Luukko[1,5,*,], Byron Drury[2], Anna Klales[3], Lev Kaplan[4], Eric J. Heller[3], and Esa Räsänen[5]**

[1]Nanoscience Center, Department of Physics, University of Jyväskylä, Jyväskylä FI-40014, Finland
[2]Department of Physics, Massachusetts Institute of Technology, Cambridge, Massachusetts 02139, USA
[3]Department of Physics, Harvard University, Cambridge, Massachusetts 02138, USA
[4]Department of Physics and Engineering Physics, Tulane University, New Orleans, Louisiana 70118, USA
[5]Department of Physics, Tampere University of Technology, Tampere FI-33101, Finland
[*]perttu.luukko@iki.fi
[**]Current address: Max Planck Institute for the Physics of Complex Systems, Dresden D-01187, Germany

S1 Solving the periodic orbits of a circularly symmetric potential in 2D

The solution of the equations of motion for a classical particle in a circularly symmetric potential $V(r)$ can be found in standard texts on classical mechanics (e.g., Chapter 3 in Ref. 1), but we summarise it here. The total energy E of the particle is the sum of kinetic and potential energy, which in polar coordinates reads (in units where mass $m = 1$)

$$E = \tfrac{1}{2}\dot{r}^2 + \frac{L^2}{2r^2} + V(r), \tag{1}$$

where L is the angular momentum, which is also a constant of motion. Solving for $\dot{r}$ from Eq. (1) gives

$$\dot{r} = \pm\sqrt{2}\sqrt{E - V(r) - \frac{L^2}{2r^2}}. \tag{2}$$

Changing variables in Eq. (2) from time to polar angle ϕ (using $\dot{\phi} = L/r^2$) and inverting gives

$$\frac{\mathrm{d}\phi}{\mathrm{d}r} = \pm\frac{L}{r\sqrt{2r^2\left[E - V(r)\right] - L^2}} := \pm\frac{L}{r\sqrt{f(r)}}, \tag{3}$$

where the function inside the square root is denoted as f. Equation (3) can be conveniently integrated to give the polar angle ϕ as a function of the radial coordinate r.

Assuming that $V(r)$ is a potential well ($V(r)$ is monotonically increasing and larger than E for large enough r) and that $L \neq 0$, the radius of the particle oscillates between two turning points $r = a$ and $r = b$, which can be solved from Eq. (2) by setting $\dot{r} = 0$. Not surprisingly, these turning points are exactly where $\mathrm{d}\phi/\mathrm{d}r$ diverges, i.e., the zeros of f in Eq. (3). Between these turning points the radial coordinate either monotonically increases or decreases, corresponding to the positive and negative solutions in Eq. (2).

When the particle completes one oscillation from $r = a$ to $r = b$ and back, the corresponding change in the polar angle (by integrating Eq. (3)) is

$$\Delta\phi = \int_a^b \frac{2L}{r\sqrt{f(r)}}. \tag{4}$$

Note that $\Delta\phi$ is completely specified by the form of the potential $V(r)$ and the values for E and L. The particle eventually returns to its starting point, i.e., the orbit is periodic, exactly when $\Delta\phi$ is a rational multiple of 2π. If $\Delta\phi = 2\pi\frac{m}{n}$ for integer n and m, after n oscillations between the radial turning points, the particle has rotated around the origin m times, returning exactly to its starting position with its original velocity, i.e., the classical oscillation frequencies are in $m\!:\!n$ resonance. If the orbit is not periodic, it is quasiperiodic.

Searching for periodic orbits (POs) for a given potential $V(r)$ and total energy E can be done conveniently by looking for zeros of the function $g(E, L, n) := \sin\left(\tfrac{1}{2}n\Delta\phi\right)$. This can be done by any standard root-finding method, using L as the free variable, and some set of discrete choices for the integer n. This gives values of L which correspond to a PO, and the integers n and m, which give the shape of the orbit.

The procedure is especially simple if $V(r)$ is a homogeneous function of r. In this case different total energies differ only by a scaling factor in space and time, which means the PO shapes do not depend on E. Moreover, for $V(r) \propto r^a$ with integer a, the function f in Eq. (3) is a polynomial, which makes it particularly easy to find the turning points for moderate values of a.

As an illustration, the shortest POs for some small integer values of a are given in Table S1. Most importantly, the five-pointed star with $m/n = 2/5$ appears at $a = 5$, and the next jump to a simpler shortest PO occurs at $a = 8$ with the birth of the triangle orbit with $m/n = 1/3$. The POs of power-law potentials are also studied in a more indirect way in Ref. 2.

Supplementary Table S1. Periodic orbits for $V(r) \propto r^a$ up to $n = 15$. Table entries are aligned by n to highlight how new PO shapes appear with increasing a.

a	Periodic orbits as values of m/n									
1			4/7		5/9	6/11		7/13		8/15
2	1/2 (harmonic oscillator – a very special case)									
3						5/11		6/13		7/15
4			3/7		4/9	5/11	5/12	6/13		7/15
5		2/5	3/7		4/9	5/11	5/12	{5, 6}/13		7/15
6		2/5	3/7	3/8	4/9	{4, 5}/11	5/12	{5, 6}/13	5/14	7/15
7		2/5	3/7	3/8	4/9	{4, 5}/11	5/12	{5, 6}/13	5/14	7/15
8	1/3	2/5	3/7	3/8	4/9	{4, 5}/11	5/12	{5, 6}/13	5/14	7/15
9	1/3	2/5	3/7	3/8	4/9	{4, 5}/11	5/12	{4, 5, 6}/13	5/14	7/15

S2 Extracting scars with a wave-packet "scarmometer"

As explained in the main article, for ordinary scars a wave packet initialised on a PO can be used to locate eigenstates that are scarred by that particular orbit, since the scarred eigenstates will have large overlaps with the wave packet. Similarly a wave packet initialised on a specific PO of the unperturbed system can be used to isolate scars with that particular orientation in the perturbed system. This is illustrated in Supplementary Fig. S1, which uses the wave packet used in Fig. 2 to isolate scarred eigenstates with the same orientation as the $n = 2720$ example scar used throughout the main article. This illustration also shows how several scarred eigenstates contribute to the recurrences of a single wave packet.

S3 Scars of other resonances in homogeneous potentials

The five-pointed star orbit in the r^5 potential is in some ways a natural "sweet spot" for scarring. The 2 : 5 resonance produces tight near-degenerate resonant sets, because moving from one resonant state to the next involves the exchange of a small number of quanta, making the degeneracy approximation better.

The five-fold symmetry also gives more room for visually distinct preferred orientations, as opposed to, e.g., an 11-pointed star. Moreover, the existence of a self-crossing point in the classical orbit makes the probability density in a scar less uniform, which helps the localisation of the scars as they will pin more strongly to impurities near the self-crossing point. This gives the five-pointed star some advantage over the two simpler resonances, 1 : 3 and 1 : 4.

In other homogeneous potentials, scarring due to classical resonances exists analogously to the r^5 case. With other choices of the exponent in the unperturbed potential, different resonances exist, as summarised in Table S1. This is reflected in the shape and abundance of scars. Figure 1 includes examples of scars in a r^8 potential.

The integer exponent $a = 3$ is unusual in that its simplest non-trivial resonance is 5 : 11. With coefficients so large the near-degeneracy in the resonant sets becomes poor, and the scars become rare and distorted. In fact, at this limit the near-degeneracy due to resonant sets might compete with purely accidental near-degeneracies. An even more extreme absence of short non-trivial classical resonances can be found with non-integer exponents close to 2.

S4 Scars in a non-homogeneous potential

While a homogeneous potential function simplifies the classical PO structure, it is not necessary for scarring. Other forms of the unperturbed potential also have quasi-degenerate sets of eigenstates due to classical resonances, and thus local impurities will cause scars. Figure 1 contains an example of a five-pointed scar eigenstate for $V(r) \propto \cosh(r) - 1$. In this case, the parameters of the perturbation are such that each Gaussian bump has amplitude $M = 10$, FWHM of 0.353, and the average density of bumps is 2.4 bumps per unit square.

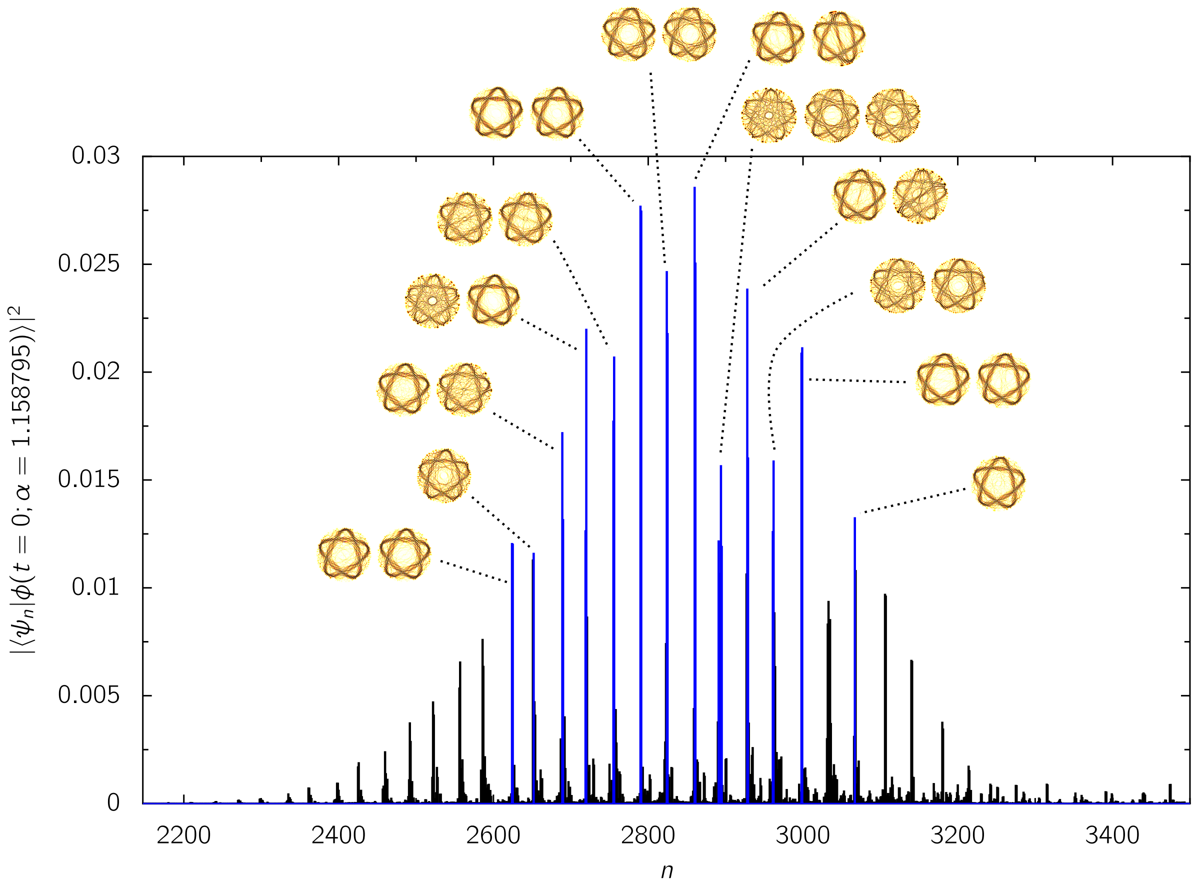

Supplementary Figure S1. Extracting scars with a specific orientation with a wave packet "scarmometer". The histogram in the background shows the basis decomposition of the wave packet used in Fig. 2 in the eigenstates of the perturbed system. Scarred eigenstates show up as prominent peaks in this decomposition. For some of the strongest peaks (marked in blue) the corresponding eigenstate is also shown, highlighting that they are all scarred to a varying degree, and that the orientation of the five-pointed scar is the same.

To provide more proof that similar scarring also exists for a non-homogeneous potential, Supplementary Fig. S2 shows wave-packet recurrences as a function of the wave-packet orientation angle, and Supplementary Fig. S3 shows eigenstates contributing to the recurrences of the strongest preferred orientation.

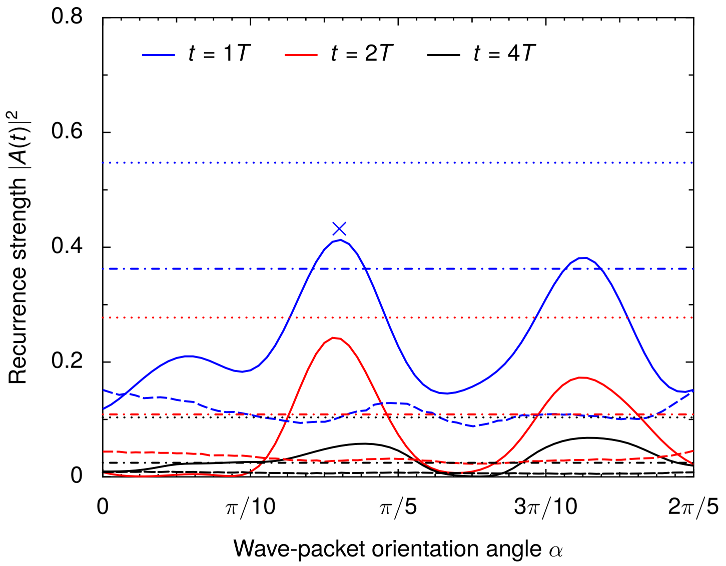

Supplementary Figure S2. Plot equivalent to Fig. 4 except for a non-homogeneous unperturbed potential $V(r) \propto \cosh(r) - 1$, with impurity parameters described in the text. The wave-packet was initialised on a 2:5 PO with energy $E = 200$.

S5 Generality of the results for different random realisations of the impurities

We have not attempted to specify quantitatively how common the scars or preferred orientations are among all random realisations of the impurity locations. To illustrate that they are not a rare occurrence by any means, Supplementary Fig. S4 shows plots equivalent to Fig. 3, but with 10 randomly selected realisations of the impurity potential, each with the same parameters. The realisations were generated by seeding the random number generator (RNG) in `itp2d`[3] with integers from 1 to 10.

While the preferred direction branches are not always as clear as in the example realisation used in the main article, preferred orientations and accompanied strongly scarred states (signified by the large overlap with the wave packet) are present in all the shown cases.

The RNG seed used for the realisation discussed in the main article is 20141010. In the interest of full reproducibility, the version of `itp2d` used was `1.0.0-7-gd3c0454`, with command line parameters:

```
itp2d --rngseed 20141010
  -F abschange(1e-3) -T absstdev(1e-3,5e-4)
  -l 11 -s 300 -e 0.01 -d 12 -t 6
  --noise impurities
  --impurity-distribution "uniform(2.0)"
  --impurity-type "gaussian(24, 0.1)"
  -n 4000 -N 5000
  -p "poweroscillator(5)"
  --maxsteps 50 --recover -D 2
```

References

1. Goldstein, H. *Classical Mechanics* (Addison-Wesley, 1980), second edn.
2. Reynolds, M. A. & Shouppe, M. T. Closed, spirograph-like orbits in power law central potentials. *ArXiv e-prints* **1008.0559** (2010). 1008.0559.
3. Luukko, P. J. J. & Räsänen, E. Imaginary time propagation code for large-scale two-dimensional eigenvalue problems in magnetic fields. *Comput. Phys. Commun.* **184**, 769–776 (2013).

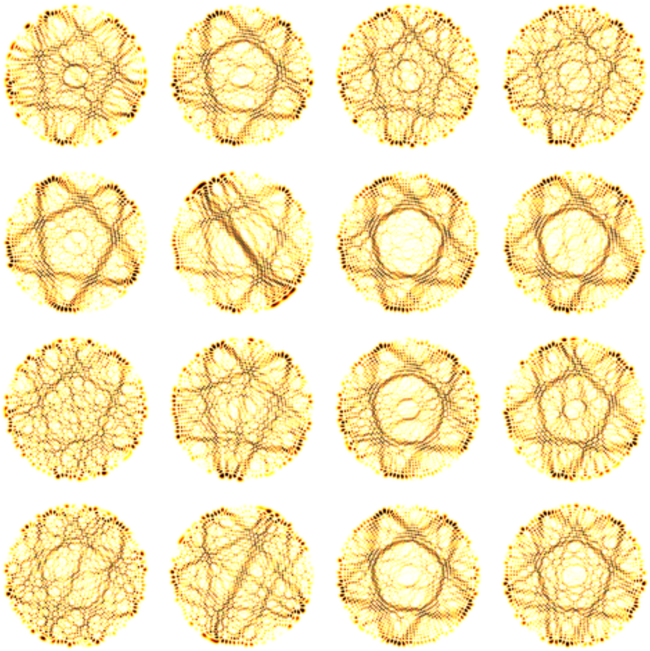

Supplementary Figure S3. Most prominent eigenstates in the decomposition of the wave-packet in Supplementary Fig. S2 at the most strongly recurring orientation (marked with a blue cross in Supplementary Fig. S2).

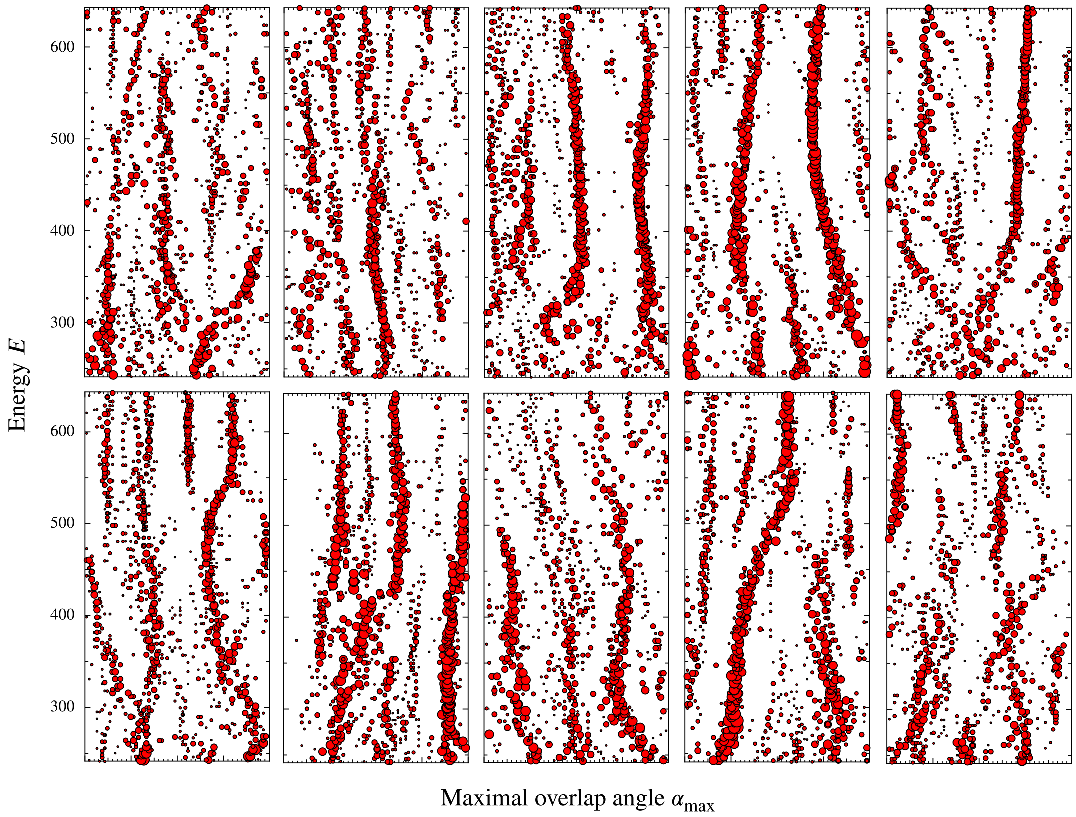

Supplementary Figure S4. Montage of plots equivalent to Fig. 3 except than with ten other random realisations of the impurity positions. Each case shows branches of preferred orientations consisting of scarred eigenstates, although the strength of the branches and their stability with respect to eigenstate energy varies. In all plots the range of α_{max} is from 0 to $2\pi/5$.
